# Mitochondrial Genetic Background May Impact Statins Side Effects and Atherosclerosis Development in Familial Hypercholesterolemia

**DOI:** 10.3390/ijms24010471

**Published:** 2022-12-28

**Authors:** Eduardo Ruiz-Pesini, María Pilar Bayona-Bafaluy, Teresa Sanclemente, José Puzo, Julio Montoya, David Pacheu-Grau

**Affiliations:** 1 Departamento de Bioquímica, Biología Molecular y Celular, Universidad de Zaragoza, 50009 Zaragoza, Spain; 2 Departamento de Bioquímica, Biología Molecular y Celular, Universidad de Zaragoza, 50013 Zaragoza, Spain; 3 Instituto de Investigación Sanitaria (IIS) de Aragón, 50009 Zaragoza, Spain; 4 Centro de Investigaciones Biomédicas en Red de Enfermedades Raras (CIBERER), 28029 Madrid, Spain; 5 Instituto de Biocomputación y Física de Sistemas Complejos (BIFI), Universidad de Zaragoza, 50018 Zaragoza, Spain; 6 Facultad de Ciencias de la Salud y el Deporte, Universidad de Zaragoza, 22002 Huesca, Spain; 7 Unidad de Lípidos, Servicio de Bioquímica Clínica, Hospital General Universitario San Jorge de Huesca, 22004 Huesca, Spain; 8 Departamento de Medicina Universidad de Zaragoza, Universidad de Zaragoza, 50009 Zaragoza, Spain

**Keywords:** familial hypercholesterolemia, mtDNA, mitochondria, CoQ_10_

## Abstract

Heredity of familial hypercholesterolemia (FH) can present as a dominant monogenic disorder of polygenic origin or with no known genetic cause. In addition, the variability of the symptoms among individuals or within the same families evidence the potential contribution of additional factors than monogenic mutations that could modulate the development and severity of the disease. In addition, statins, the lipid-lowering drugs which constitute the first-line therapy for the disease, cause associated muscular symptoms in a certain number of individuals. Here, we analyze the evidence of the mitochondrial genetic variation with a special emphasis on the role of CoQ_10_ to explain this variability found in both disease symptoms and statins side effects. We propose to use mtDNA variants and copy numbers as markers for the cardiovascular disease development of FH patients and to predict potential statin secondary effects and explore new mechanisms to identify new markers of disease or implement personalized medicine strategies for FH therapy.

## 1. Introduction

Familial hypercholesterolemia (FH) is a disease with a genetic origin that promotes atherosclerotic cardiovascular disease and leads to increased and premature mortality [1]. Recent studies have calculated the frequency of its heterozygous form (HeFH) ranging from 1/313 to 1/200 [2,3,4], and, therefore, it has been considered the most common autosomal dominant genetic disorder affecting humankind [5]. Most patients carry mutations in the classical associated FH genes *LDLR* (low-density lipoprotein receptor), *APOB* (apolipoprotein B) or *PCSK9* (proprotein convertase subtilisin/kexin type 9) and some show rare alleles in the minor genes *APOA5* (apolipoprotein A-V), *ABCG5* (ATP-binding cassette sub-family G member 5), *ABCG8* (ATP-binding cassette sub-family G member 8), *LIPA* (lysosomal acid lipase) and *STAP1* (signal transducing adaptor family member 1). Interestingly, a good number of cases show either a combination and accumulation of multiple common variants (polygenic trait) or no known genetic cause associated with FH [6]. In addition, 80% of common DNA variants that would predispose an individual to coronary artery disease are not related to cholesterol pathways but to others like inflammation, vascular tone, cellular proliferation, and many other pathways yet to be discovered [7].

In a similar way, there is a big variability described in symptoms related to FH caused by mutations in the same gene, even in relatives from the same family sharing the same mutations but, in some cases, showing no FH phenotype or surviving without cardiovascular adverse events [8,9,10]. This variation is probably a combination of environmental factors and common DNA variants that interact with primary FH pathological mutations, influencing its penetrance and expressivity.

Mitochondria are metabolic organelles whose main function is to convert nutrients into cellular energy in the form of ATP [11]. They are also importantly involved in lipid metabolism and homeostasis. Mitochondria convert fatty acids into energy, performing beta-oxidation. In addition, the mevalonate pathway converts acetyl-CoA (CoA) into different metabolically relevant precursors, like Coenzyme Q_10_ (CoQ_10_) and cholesterol among others [12,13]. Interestingly, genetic defects in *LDLR* may abolish LDL binding to its receptor. This binding controls the activity of HMGCR (3-hydroxy-3-methyl-glutaryl-coenzyme A reductase), the rate-limiting enzyme of cholesterol biosynthesis, therefore explaining the increased intracellular and plasma cholesterol levels in FH patients [14,15]. Moreover, increased cholesterol levels derived from LDL defective binding result in decreased CoQ_10_ de novo synthesis, total CoQ_10_ levels, and reduced gene expression of enzymes involved in CoQ_10_ biogenesis. Thus, mutations causing FH may cause dysregulation of the mevalonate pathway and a reduction in CoQ_10_ synthesis and lead to secondary mitochondrial dysfunction [14]. Another interesting mechanism regarding CoQ_10_ levels and HF is the downregulation of HMGCR through statins. These drugs are the chosen therapy for HF and have been shown to decrease mortality and coronary artery disease by drastically lowering circulating lipids [16,17,18]. However, an important number of patients treated with these drugs (7–19%) suffer from muscular symptoms derived from statin treatment (SAMS) [19]. Interestingly, mevalonate pathway dysregulation, resulting in decreased CoQ_10_ levels and concomitant secondary mitochondrial dysfunction, has been proposed to be causing SAMS [20,21,22]. Therefore, CoQ_10_ supplementation has been proposed as a complementary statin therapy in some patients in order to improve SAMS. Nevertheless, there is some controversy about the efficiency of these treatments, since some studies were able to find significant improvement of SAMS upon CoQ_10_ supplementation [23,24,25], whereas other studies were not able to identify any positive effect [26,27]. One relevant factor is CoQ_10_ bioavailability and absorption due to its low water solubility [28,29]. Therefore, many studies trying to address the effect of CoQ_10_ supplementation have been not able to estimate the real amount of the compound that reaches target tissues [30]. Interestingly, a genetic component has been postulated to modulate SAMS [31]. Here, we will introduce some interesting aspects of how mitochondrial genetic background may impact symptom variability in FH and muscle deficiency associated with statins therapy.

## 2. Mitochondrial DNA Genetic Variation May Contribute to FH Development

One of the most interesting features of mitochondria is that they contain their own genome, the mitochondrial DNA (mtDNA), which is a small circular genome present in a variable copy number in almost all human cells. MtDNA encodes for 13 proteins, which are components of the oxidative phosphorylation system (OXPHOS). OXPHOS complexes are located in the inner mitochondrial membrane and are responsible for the production of most cellular energy in form of ATP. In addition, mtDNA encodes for 22 tRNAs and 2 rRNAs, which are key elements involved in the synthesis of the 13 mtDNA-encoded OXPHOS proteins, inside mitochondria. MtDNA mutations have been associated with mitochondrial diseases, a severe and heterogenous group of disorders often caused by a deficient ATP synthesis derived from OXPHOS malfunction [32,33]. Interestingly, mtDNA variation has not only been linked to mitochondrial diseases. Many mtDNA genetic variants are ancient mutations that survived and expanded in populations. These polymorphisms are relatively frequent in human populations and define clusters of phylogenetically related mitochondrial genotypes (or mitochondrial haplogroups) [34]. The effect of population mtDNA variants has been studied during the last few decades, and it has been associated with many relevant pathophysiological conditions, such as antibiotic therapy side-effects, survival to sepsis, sperm motility or Parkinson’s disease [35,36,37,38,39,40]. Recently, several studies have tried to find a connection between mtDNA and the missing heritability of some FH cases and other related pathologies. Vilne et al. examined the association between mitochondrial genome variation and coronary artery disease. They studied 265 mitochondrial single nucleotide variants (mt-SNVs) in approximately 500,000 UK general population individuals. According to their clinical phenotype, individuals were divided into two different groups of coronary artery disease patients, termed SOFT (angina and chronic ischemic heart disease) and HARD (myocardial infarction and or revascularization) and a control group. They found a higher prevalence of variants m.295C > T in the control region, m.12612A > G, m.12372 G > A in the gene *MT-ND5* and m. 11467A > G in the gene *MT-ND4* in HARD patients. In the same way, SOFT patients showed a higher prevalence of variants m.10400C > T in the gene *MT-ND3*, m.11251A > G in the gene *MT-ND4*, m.15301G > A and m.15452C > A in the gene *MT-CYB*. Interestingly, an important number of coronary artery disease patients (51.2% in HARD and 44.8% in SOFT) presented with hypercholesterolemia in this study. Unfortunately, no other genetic information is provided to address if FH causative mutations may be contributing together with mtDNA variants to the development of coronary artery disease [41]. In addition, several studies have analyzed the influence of mtDNA copy number in cardiovascular diseases. For example, Ashar and coworkers measured mtDNA copy numbers in around 22,000 samples derived from 3 different retrospective studies that recorded cardiovascular accidents for more than 13 years and found an inverse association between mtDNA copies and cardiovascular events. In addition, mtDNA levels were proposed to be used as a prediction tool for cardiovascular disease risk [42]. A prospective study using approximately 300,000 samples from UK general population measured mtDNA copy number and registered for approximately 12 years of cumulative incidence of coronary artery disease or heart failure and found an association of lower mtDNA copies with a higher risk of coronary artery disease [43]. In order to address the interesting hypothesis that a decreased mtDNA copy number is related to a higher risk of cardiovascular disease through a higher disposition to develop cardiometabolic conditions (such as obesity, higher lipids or glucose levels in blood… etc.), which are known risk factors for cardiovascular accidents, Vasan et al. studied the levels of mtDNA and its association with different cardiometabolic disease traits in approximately 400,000 individuals of different ethnic origin. Interestingly, they found significant correlations between mtDNA copy numbers and obesity, hypertension, diabetes, and hyperlipidemia. The authors proposed that reduced levels of mtDNA results in decreased energy production and this may be involved in the development of a set of conditions than will increase the risk of a cardiovascular event [44].

## 3. Mitochondrial Genetic Variation in Nuclear Genes and FH Development

CoQ_10_ is a lipid molecule that transfers electrons within the electronic transfer chain being therefore essential for ATP production inside mitochondria [45]. This lipid molecule has been shown to effectively prevent the oxidation of plasma circulating LDL particles [46]. Since oxidized LDL seems to be critical for the signaling cascade resulting in the formation of atherosclerosis, it is plausible that CoQ_10_ levels play a role in the development of cardiovascular disease in FH individuals. Primary CoQ_10_ deficiencies, derived from mutations in genes involved in the synthesis of this compound, show decreased CoQ_10_ levels in affected tissues and cause mitochondrial dysfunction [47]. In addition, with other metabolic genetic disorders, such as phenylketonuria (PKU) or mucopolysaccharidoses (MPS), patients have been described to present with decreased CoQ_10_ levels [48]. However, it is highly unlikely that any of these genetic conditions that lower CoQ_10_ levels occur concomitantly with FH-causing mutations. Therefore, it is much critical to understand how CoQ_10_ levels may vary among normal individuals to understand its potential role in atherosclerosis development in FH. Interestingly, CoQ_10_ normal values have been shown to be significantly different among individuals due to their racial origin [49,50]. Therefore, several studies have tried to identify the genetic determinants of this observed variation. The study of Fischer and coworkers analyzed the relationship between SNPs in genes involved in the biosynthesis, reduction, and metabolism of CoQ_10_ at the basal level and after supplementation in 54 healthy volunteers. This study identified a homozygous SNP in the *NQO1* gene, responsible for generating and maintaining the reduced state in quinones, that correlated with reduced CoQ_10_ levels and therefore could participate in abnormal coenzyme metabolism, Interestingly, this effect was not observed after supplementation. In addition, the authors found a correlation between Apolipoprotein E alleles E4/E4 with higher CoQ_10_ levels after supplementation. This allele has been associated with oxidative stress and inflammation, and E4/E4 individuals seem to be more responsive to dietary CoQ_10_ supplementation [51]. In addition, genome-wide association studies (GWAS) have identified an association between CoQ_10_ levels and SNPs in *COLEC12* and *NRXN-1* genes, and, although neither of them are directly related to mitochondrial function, they have been linked to neurological diseases like Alzheimer’s disease, where mitochondria are known to play an important role [52]. Surprisingly, these studies were unable to find genetic variants directly involved in the mitochondrial CoQ_10_ biosynthetic pathway that could explain the observed variability in the coenzyme levels.

As already mentioned above, CoQ_10_ supplementation has been used to avoid undesired secondary effects from statin therapy [23]. In order to identify dietary or genetic determinants that could explain the variable circulating levels of this molecule after supplementation, Takahashi et al. found 4 different SNPs in genes involved in cholesterol metabolism (*CYP7A1*), CoQ_10_ absorption at the intestine (*NPC1L1*), CoQ_10_ efflux (*ABCB1*) and cellular CoQ_10_ uptake (*CD36*) that were involved in the variability observed in CoQ_10_ levels in serum after long-term supplementation in women [53,54]. Thus, SNPs in these genes and others may also influence CoQ_10_ bioavailability from dietary sources and derive into different physiological levels of the coenzyme that could differentially impact atherosclerosis development in FH patients.

In the same line of evidence with the reported phenotypical variability among FH patients and relatives, recent studies have shown that FH patients have a different predisposition to suffer cardiovascular disease events. To identify the genetic component of this susceptibility, Paquette et al. analyzed the relationship between genetic risk scores for coronary artery disease and cardiovascular disease events in FH patients carrying a known pathologic mutation and found a strong association between these 2 parameters. Therefore, they could demonstrate that common genetic variants are able to modify the phenotypical expression of FH [55]. Interestingly, the SNPs used to implement the genetic risk score for coronary artery disease derived from previous GWAS that identified variants robustly associated with coronary artery disease risk. Indeed, SNPs within mitochondrial genes, like intronic regions of the GTPase *NOA1*, required for mitochondrial protein synthesis have been associated with the risk of coronary artery disease [56,57,58]. In the same line, risk alleles for the same condition have been identified in *MRPS6*, a component of the mitochondrial ribosome and therefore critical for mitochondrial protein synthesis and cellular bioenergetics [59,60,61]. In addition, SNPs in *OCTN1* (*SLC22A4*), a cation/carnitine transporter postulated to be localized inside mitochondria have been also linked to coronary artery disease [56,62].

## 4. Mitochondrial Genetic Landscape of SAMS

Statins are widely prescribed as first-line therapy for the prevention of coronary artery disease in FH patients. However, a big variability has been described in the effectiveness of these drugs. Interestingly, genetic variation has been proposed as an explanation for this variability and many different genes related to the pharmacokinetics and pharmacodynamics of statins have been investigated [63,64]. Indeed, the contribution of mtDNA variants (haplogroups) to statin efficacy was addressed among the Chilean population of Amerindian origin. Nevertheless, no significant association was found between pre- and post-treatment lipid levels and mitochondrial genetic background [65]. An important burden of statin treatment is the development of muscle symptoms derived from the treatment (SAMS) in an important percentage of patients [19]. Following the same reasoning, it is plausible that different secondary effects from statin therapy are determined by genetic variation among individuals. One of the hypotheses proposed to explain the muscular symptoms is a decreased level of CoQ_10_ and a concomitant mitochondrial dysfunction [20,21,22]. Apart from those mechanisms influencing biosynthesis, bioavailability and metabolism of CoQ_10_ (already discussed in the previous section), different sources of mitochondrial genetic variation might be considered to explain statin secondary effects. One recent study identified the variant m.12630G > A to correlate with a higher risk for myalgia in Chinese patients with coronary artery disease treated with statins [66]. This mutation is located in the *MT-ND5* gene (Complex I subunit) and mechanistic details about how this synonymous variant could influence the prevalence of SAMS are yet to be investigated. The study by Stringer and coworkers analyzed the levels of mtDNA in patients treated with statins that developed muscular symptoms compared to those who did not suffer side effects derived from statins. Interestingly, patients with muscle symptoms showed significantly lower levels of mtDNA than non-affected patients. However, due to the retrospective nature of the study, the authors could not establish whether the mtDNA decrease was due to statin therapy or a previously existing condition that would predispose those patients to SAMS [67]. In both cases, mtDNA copy variation may account for different susceptibility to statin therapy.

A different hypothesis has been recently proposed to explain SAMS. Schirris et al. proposed that statin lactones (form causing more cytotoxic effects) could bind to the Q_o_ site on Complex III and inhibit electron transfer and respiration. This inhibition seems to be dependent on the available reduced CoQ_10_ and therefore could be influenced by other susceptibility factors. In addition, authors demonstrate that individuals with impaired Complex III activity due to genetic mutations were more sensitive to statin lactones cytotoxicity than controls [68]. Therefore, genetic variation in Complex III genes might also contribute to SAMS development. Interestingly, SNPs within the Q_o_ site of the cytochrome b gene (*MT-CYB*) define some important mtDNA haplogroups. For example, amino acid changes A122T characterize the African haplogroup L2bc, G251S Asian haplogroup G1 and the changes T158A and D171N define the frequent European haplogroups U5a1 and J2 respectively. Variants in the Q_o_ site have been proposed to modify mitochondrial coupling efficiency and therefore ATP, heat and ROS production [69]. Following this line of evidence, these variants have been associated with phenotypical effects. For example, haplogroup U5a (including U5a1) and J (including J2) were elevated among HIV-1 infected patients that developed faster AIDS and eventually died. In addition, the change in the G251S defining haplogroup G1 has been associated with obesity in the Japanese population [39,70]. Variant T158A has also been found to be more frequent in individuals with epilepsy and maternal relatives with epilepsy and hearing loss [70]. If common mtDNA variants within the Q_o_ site of complex III, have been associated with functional effects, it is tempting to speculate that they could influence the potential binding of statin lactones to complex III and therefore modulates electron transfer and ATP production inhibition caused by statins, giving an explanation for the different susceptible individuals to SAMS.

## 5. Non-Genetic Variables to Be Considered

Not only genetic determinants may influence the progression and features of FH patients. Environmental factors may also play a role by interacting with primary pathogenic FH mutations. Nutrition is the most important environmental factor acting on gene expression during the entire life span of human beings [71]. Interestingly, FH patients from different countries showed the different risks of cardiovascular disease associated with different dietary habits. The adherence to the Mediterranean diet inversely correlated with dyslipidemia and low-grade inflammation biomarkers even after adjustments for strong cofounders like pharmacologic LDL lowering therapies. These results highlight the potential role of dietary patterns in dyslipidemia and inflammation profiles in FH caused by autosomal dominant mutations [72].

In addition, recent studies about the dietary habits of patients with familial hypercholesterolemia showed that FH-diagnosed patients exhibited a healthier life and nutritional habits than their non-FH relatives, probably motivated by the awareness of their genetic condition [73]. Therefore, dietary habits and adherence to a healthier lifestyle are variables that may influence the development and progression of FH and must be taken into account when studying genetic determinants of the disease.

## 6. Future Directions

FH has been typically classified as a dominant monogenic disorder. However, in many cases, whether hereditability could be traced back to polygenic traits or genetic causes remained unknown. In addition, the variability of the symptoms among individuals or within same families evidence the potential contribution of additional factors than FH causing mutations that could modulate the development and severity of the disease. Here, we have described several pieces of evidence, detailing how mtDNA variants and copy number might contribute to coronary artery disease and therefore influence the progression of FH to cardiovascular accidents. In addition, we have discussed the potential role of CoQ_10_ in atherosclerosis development and presented some genes that could affect its regulation and bioavailability. Although these genes had no direct effect on mitochondria, the tight relation between CoQ_10_ and mitochondrial respiration may result in different antioxidant capacity of CoQ_10_ and therefore mitochondrial genetic variation (either in mtDNA or nuclear-encoded genes) resulting in different OXPHOS performance would probably impact CoQ_10_ role as an antioxidant. Indeed, another important player to be accounted are the reactive oxygen species (ROS) produced in the respiratory chain. Any mitochondrial genetic variation that would increase ROS production, may overcome the protective effect of CoQ_10_ or other antioxidant systems and potentially increase oxidized LDL to start the formation of atherosclerotic plaque [74]. Finally, we showed that variants in mitochondrial genes have been associated with polygenic traits causing FH or coronary artery disease. Understanding the molecular consequences of these variants will provide information of how different mitochondrial pathways may influence the development and severity of the disease.

We have also explored different alternatives to explain the different susceptibility to statin therapy that could derive in the appearance of SAMS. MtDNA levels could offer a protective effect, to the secondary mitochondrial dysfunction produced by statin therapy. Indeed, mtDNA levels have been shown to contribute to different pathological conditions [75]. In addition, genetic variants in mtDNA within Q_o_ site in Complex III, (*MT-CYB* gene) where CoQ_10_ binds to be oxidizied, could influence the binding of lactone form of statins that have been shown to inhibit Complex III electron transfer and respiration. The population frequency of these variants could explain the appearance of muscular symptoms in a certain percentage of individual treated with statins.

We propose to analyze mtDNA variation and mtDNA levels in FH patients as a marker for development to cardiovascular disease. In addition, variants within Q_o_ site could be used to potentially predict secondary effects of statins therapy. Cellular studies will be useful to analyze the interaction between *MT-CYB* genetic variants and statins to address if different statins would affect different individuals with certain variants in order to implement personalized medicine strategies for FH therapy.
